# Enhance therapeutic efficacy of BiTE (HER2/CD3) for HER2- positive tumors through *in vivo* expression

**DOI:** 10.1016/j.ijpx.2025.100375

**Published:** 2025-08-14

**Authors:** Huifang Zong, Xi Li, Yunxia Li, Lei Wang, Yali Yue, Jie Chen, Yong Ke, Pameila Paerhati, Lei Han, Yijia Li, Jianwei Zhu, Baohong Zhang

**Affiliations:** a Engineering Research Center of Cell & Therapeutic Antibody, Ministry of Education, China, School of Pharmacy, Shanghai Jiao Tong University, Shanghai 200240, China; b Jecho Institute, Co. Ltd., Shanghai 200240, China; c Yunnan Suns Regenerative Medicine Engineering Co. Ltd., Kunming 650106, China

**Keywords:** BiTE, MSCs, Anti-tumor, Pharmacokinetic

## Abstract

Bispecific T-cell engagers (BiTEs) are small-molecule antibodies that exhibits potent tumoricidal activity but suffer from a short plasma half-life. Mesenchymal stromal cells (MSCs) represent promising delivery vehicles for sustained therapeutic protein expression. In this study, we used human umbilical cord blood-MSCs (hUC-MSCs) as a delivery system to to secrete HER2/CD3 BiTE antibodies, thereby addressing the pharmacokinetic limitations of conventional BiTE therapies. HER2 amplification and overexpression are observed in multiple solid tumors, making it a potent target for anti-cancer therapies. Therefore, we constructed a BiTE targeting HER2 and CD3 as a model. *In vitro* efficacy, both MSCs and MSC-BiTE supernatants could induce significant cell death in BT474 and NCIN87 cells. *In vivo*, MSC-BiTE inhibited tumor growth in NCIN87 xenograft model. Furthermore, MSC-BiTE elevated the plasma levels of BiTE (HER2/CD3) antibody. Therefore, MSC-BiTE may be used as an efficient therapeutic agent for HER2-positive cancers.

## Graphical abstract

**Figure:**
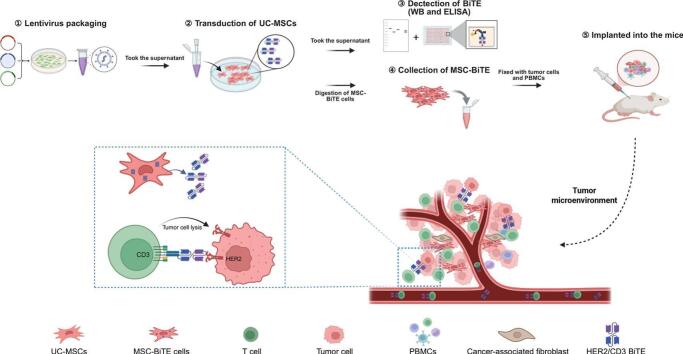

## 1 Introduction

Members of the epidermal growth factor receptor family (*Erbb* family) are potent mediators of cell growth and development (Hynes and Lane, 2005). Amplification and overexpression of *ERBB2* (also known as HER2), have been extensively documented in various human solid tumors (Komoto et al., 2009), including breast cancer (Yaziji et al., 2004), ovarian cancer (Vermeij et al., 2008), gastric cancer (Garcia et al., 2003), and salivary gland tumors (Press et al., 1994). Tumor cells manifest a higher abundance of HER2 molecules on their surface compared to normal cells. In addition, high HER2 levels are closely related to poor prognosis in patients with HER2-overexpressing cancers, particularly in cases of breast cancer (Wang and Hung, 2001).

The HER2 antigen is a potent target for anti-cancer therapies. Specifically, its location on the surface of cells makes it a potent immunotherapy target. Different types of drugs targeting the HER2 protein have been developed, including monoclonal antibodies, antibody drug conjugates and bispecific antibodies. Some drugs have been approved by the FDA, such as trastuzumab, pertuzumab and trastuzumab deruxtecan. Additionally, multiple anti-HER2 bispecific antibodies, particularly HER2/CD3 bispecific antibodies are currently under investigation in preclinical and clinical studies to evaluate their therapeutic efficacy (Kiewe et al., 2006; Yu et al., 2019).

Bispecific T-cell engager (BiTE), a 55-KDa small-molecule antibody, can augment anti-tumor effects through recruiting effector T cells (Hoffman and Gore, 2014). The main characteristics of BiTE antibodies distinguishing them from other bispecific antibody constructs include their ability to redirect target cell lysis with reduced the T cell numbers (Baeuerle and Reinhardt, 2009; Huehls et al., 2015), activate T cells upon target cell recognition, and enable serial lysis by activated T cells at low effector-to-target (E:T) ratios (Nagorsen and Baeuerle, 2011). Compared to IgG antibodies, small BiTE molecules demonstrate superior solid tumor penetration. Blinatumomab, a CD19/CD3 BiTE antibody, is an approved drug showing positive therapeutic effects in patients with non-Hodgkin's lymphoma. However, BiTE antibody applications are limited by their short serum half-life of a few hours, requiring continuous intravenous infusion, often administered *via* portable minipumps in clinical settings. This limitation results in suboptimal drug delivery to tumor sites, highlighting the need for improved targeted delivery systems.

Genetically modified mesenchymal stromal cells (MSCs) represent promising delivery vehicles for protein expression in tumor treatment (Chulpanova et al., 2018; Rustad and Gurtner, 2012). The four key advantages of MSCs used to treat tumors are low immunogenicity, ease of isolation and expansion, infected sites tropism, and genetic modifiability (Yan et al., 2013). MSCs can be genetically engineered to express tumor suppressor genes with anti-cancer activity and cytokines; and are currently being evaluated in numerous preclinical and clinical studies (Marini et al., 2017; Studeny et al., 2004; Zhang et al., 2017). For example, studeny et al. reported that MSC-interferon (IFN)-β cells inhibits pulmonary metastasis growth, potentially through the MSC secreting IFN-β in the tumor microenvironment (Studeny et al., 2004). Additionally, MSCs show promise as delivery vehicles for oncolytic viruses. Du et al. reported that MSCs carrying various oHSV variants (MSC-oHSV) selectively target metastatic lesions, significantly improving survival in mouse models of brain tumors (Du et al., 2017). Several genetically modified MSCs, including MSC-IFN-β, MSC-TRAIL, and GX-051, are undergoing clinical trials (ClinicalTrials.gov) for tumor treatment.

It is noteworthy that various studies have reported both tumor-promoting and -suppressing effects of MSCs (Khakoo et al., 2006; Li et al., 2015; Lin et al., 2019; Nowakowski et al., 2016). A previous study reported that MSCs enhanced tumor cell growth with MSC treatment compared to controls, potentially through MSC differentiation into tumor-associated fibroblasts (Suzuki et al., 2011). In contrast, another study reported that the risk of malignant transformation and consequent tumor formation subsequent to MSC transplantation is minimal and primarily theoretical (Prockop et al., 2010). In another study, MSCs inhibited tumor cell proliferation (Gauthaman et al., 2012). Studies have reported anti-tumor effects of MSCs in various types of tumors, encompassing breast cancer, osteosarcoma, and ovarian carcinoma (Gauthaman et al., 2013; Yuan et al., 2018).

In this study, we developed an anti-cancer system using engineered UC-MSCs to secrete BiTE (HER2/CD3) bispecific antibodies. BiTE (HER2/CD3)-producing MSCs exhibited prolonged half-life. The therapeutic activity of BiTE (HER2/CD3) MSCs against HER2-positive cancer cells was evaluated both *in vitro* and *in vivo*. Our system demonstrated continuous BiTE secretion, potent tumoricidal activity, and extended BiTE antibody half-life. Therefore, MSC-BiTE may be an efficient therapeutic strategy for HER2-positive cancers.

## 2 Materials and methods

### 2.1 Cell lines and cell culture

FreeStyle 293F cells were purchased from Invitrogen company. NCIN87, BT474, MCF7, HepG2, and HEK293T cells were purchased from the Chinese Type Culture Collection Cell Bank. JIMT-1 cells were purchased from Procell Life Science & Technology company and cultured in McCoy's 5 A medium. HEK293T, MCF-7, and HepG2 cells were maintained in DMEM (Gibco, Carlsbad, USA), while NCIN87 and BT474 cells were grown in the RPMI-1640 medium (Gibco) supplemented with 10 % of fetal bovine serum (FBS; Gibco). MSCs were obtained from Shunxi Regenerative Medicine Co., Ltd., and cultured in a serum-free MSC medium (Youkang, Beijing, China) containing 1 % additive (Youkang, Beijing, China). Human peripheral blood mononuclear cells (PBMCs) were isolated from blood samples of healthy donors collected at Changhai Hospital (Shanghai, China). All cells were incubated at 37 °C in a humidified atmosphere containing 5 % CO_2_.

### 2.2 Production of BiTE (HER2/CD3)

**Fig. 1:**
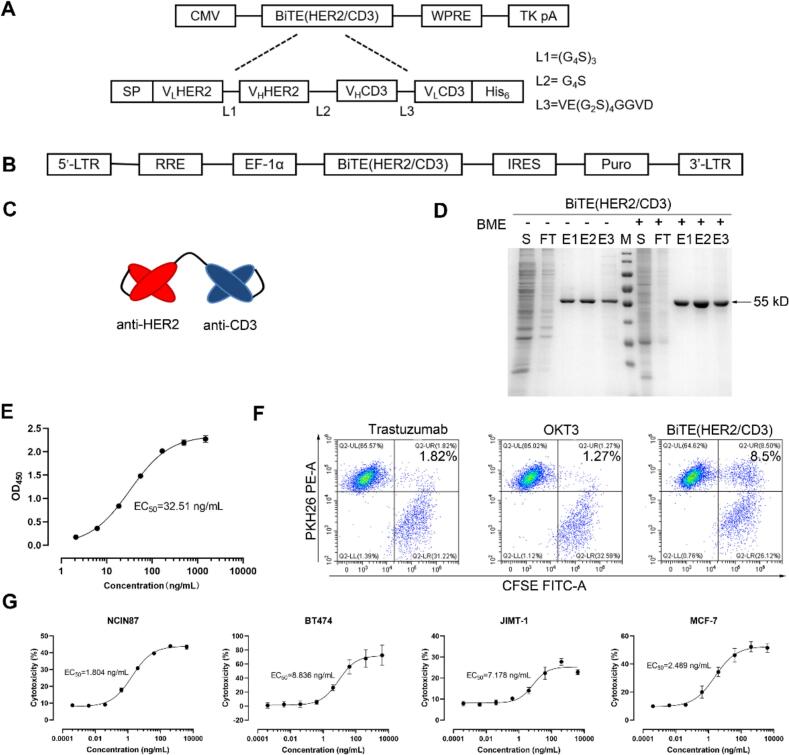
Design, production, and biological function of BiTE (HER2/CD3). (A) Schematic diagram of 293F expression vector for BiTE (HER2/CD3). CMV, CMV promoter; WPRE, woodchuck posttranscriptional regulatory element; TK pA, TK polyadenylation signal; SP, signal peptide. (B) Lentiviral expression vector for BiTE (HER2/CD3). LTR, long terminal repeats; RRE, binding site for the Rev. protein; EF-1α, EF-1α promoter; IRES, internal ribosome entry site. (C) Molecular model of BiTE (HER2/CD3). (D) SDS-PAGE analysis of the purified BiTE (HER2/CD3) with or without β-mercaptoethanol (BME) expressed by 293F cells. M, protein ladder; S, supernatants; FT, flow through; E, elution. (E) The binding affinity of BiTE (HER2/CD3) to HER2 antigen with ELISA. (F) Flow Cytometric Assessment of CD3+ Jurkat Cell Redirecting towards HER2-Positive NCIN87 Cancer Cells by BiTE (HER2/CD3), Trastuzumab and OKT3 were as negative control groups. (G) *In vitro* cytotoxicity assay of BiTE (HER2/CD3) in different HER2 expression levels cell lines through LDH release assay.

The sequences of anti-CD3 and anti-HER2 antibodies were came from OKT3 (Drugbank: DB00075) and trastuzumab (Drugbank: DB00072). *CD3* and *HER2* genes were assembled in the order of HER2VL, HER2VH, CD3VH, and CD3VL using overlap polymerase chain reaction (Fig. 1A). A His-tag was inserted into the C-terminus. The modified pcDNA3.4 vector was digested using *Nhe*I (N-terminus) and *Eco*RI (C-terminus), and the gene of interest was cloned into multiple cloning site of the vector. Subsequently, 293F cells were transfected with pcDNA3.4-BiTE (HER2/CD3) using polyethylenimine (PEI, Qifa, Shanghai, China). After seven days of transfection, when cell viability had decreased to <60 %, the supernatants were collected and purified *via* His-tag affinity chromatography (Cytiva, Shanghai, China).

### 2.3 Production of lentivirus and transduction of MSCs

The pHIV-puro vector was modified by replacing the Zsgreen gene in pHIV-Zsgreen (#18121; Addgene, Beijing, China) with a puromycin-resistance gene, followed by digestion with *Xba*I (N-terminus) and *Not*I (C-terminus). The expression plasmid pHIV-BiTE-puro was constructed by inserting the *BiTE* (*HER2*/*CD3*) gene into MCS of modified pHIV-puro vector (Fig. 1A and B).

Lentiviral particles carrying the *BiTE* (*HER2*/*CD3*) gene were packaged by co-transfection of the expression plasmid pHIV (pHIV-BiTE-puro) with a packaging plasmid (psPAX2; Addgene) and an envelope plasmid (pMD2.G, Addgene) using Lipofectamine 2000 (Invitrogen) in HEK293T cells. The pHIV-Zsgreen or pHIV-Puro as a negative control. After 48 and 72 h, supernatants containing the virus were collected and filtered through a 100-KDa ultrafiltration device (Millipore, Darmstadt, Germany) and used to transduce MSCs to generate MSC-BiTE, MSC-GFP, and MSC-EV.

MSCs were plated and cultured overnight. And then, MSCs was transduced with lentiviral particles carrying the *BiTE* (*HER2*/*CD3*) gene. GFP-infected MSCs was confirmed by fluorescence microscope at 48 h post-transduction (Nikon, Tokyo, Japan).

### 2.4 Detection of BiTE (HER2/CD3) secreting by infected MSCs

The expression of BiTE (HER2/CD3) antibodies by infected MSCs was analyzed by western blotting and enzyme-linked immunosorbent assay (ELISA). For western blotting, membranes were probed with goat anti-human His-tag antibody (Sangon, Shanghai, China) and HRP-conjugated rabbit anti-goat secondary antibody (Jackson ImmunoResearch Laboratories, Pennsylvania, USA). In the ELISA test, Human HER2 protein (HER2-hFc; Sino Biological, Beijing, China) was as coated protein and HRP-conjugated anti-His antibody (R&D System, Minnesota, USA) was as a detected antibody. BiTE (HER2/CD3) produced in 293F cell line was used as standard proteins.

### 2.5 Immunophenotype profile of MSCs

MSCs and infected counterparts (MSC-EV and MSC-BiTE) were treated with trypsin and analyzed with the Human MSC Analysis Kit (BD Biosciences, New Jersey, USA) following the manufacturer's instructions. Human positive CD markers (CD90, CD105, and CD73) and negative markers (CD45, CD34, CD11b, CD19, and HLA-DR) were assessed using a flow cytometer (BD Biosciences).

### 2.6 Adipogenic and osteogenic differentiation of MSCs

MSCs were seeded in a 6-well plate for adipogenic differentiation assay. After reaching 100 % confluency, OriCell MSC Adipogenic Differentiation Medium A and B (Cyagen, #GUXMX-90031, Guangzhou, China) were alternately used for culture for four weeks as the manufacturer's protocols. Finally, cells were cultured in medium B for seven days until the fat droplets grew sufficiently large. Then, the cells were analyzed using oil red O staining (Cyagen).

MSCs were seeded in a 0.1 % gelatin-coated 6-well plate and cultured until the degree of cell confluency reached 60–70 % to analyze osteogenic differentiation. Then, the culture medium was substituted with the OriCell MSC Osteogenic Differentiation Medium (Cyagen, #GUXMX-90021), and replaced again with a fresh medium every 2–3 d for four weeks. To visualize calcium formation, the cells were evaluated by alizarin red staining, observed under an inverted microscope (Nikon), and photographed.

### 2.7 Growth inhibitory effect of MSCs on tumor cells

MSCs were pretreated with 50 μg/mL mitomycin C (MMC; Sigma-Aldrich, St. Louis, MO, USA) for 1 h to inhibit cell proliferation. Pretreated MSCs were plated in a 96-well plate at 500 or 1000 cells/well. The tumor cells were harvested and introduced into wells in the presence or absence of MSCs at a MSC to tumor cell ratio of 1:10. The number of NCIN87 and BT474 cells was 10,000 cells/well, whereas that of MCF7 and HepG2 cells was 5000 cells/well. The growth-inhibitory effect was analyzed after 72 h using the cell counting kit (CCK)-8 assay (Dojindo Laboratories, Shanghai, China). MMC-pretreated MSCs served as controls. The suppressive impact of MSCs on the viability of tumor cells was determined using the following formula: $\text{Inhibitory rate}\;\left(\%\right)=\frac{\text{Tumor cells}–\text{Tumor cells with MSCs}+\mathrm{MSC}\;\text{control}}{\text{Tumor cells}}\times 100$.

### 2.8 T cell recruitment

The ability of T-cell recruitment about BiTE (HER2/CD3) protein was analyzed with carboxyfluorescein diacetate succinimidyl ester (CFSE) and PKH26 as described (Han et al., 2017). Antibodies trastuzumab and OKT3 were used as negative controls. Stained cells were combined in a 1:1 ratio and exposed to antibodies while being kept on ice for 1 h. After washing twice and resuspending in the FACS buffer, CFSE/PKH26 cell assembly was detected using a flow cytometer (BD Biosciences).

### 2.9 Cytotoxicity assay using BiTE (HER2/CD3)

JIMT-1, BT474, NCIN87, and MCF7 tumor cells were plated in a 96-well plate as the target cells. A gradient concentration dilution of BiTE (HER2/CD3) antibodies was introduced into the 96-well plate. And then, PBMCs was added at an E:T ratio 10:1. Following a 20-h incubation, cytotoxicity was evaluated utilizing the CytoTox 96 Non-Radioactive Cytotoxicity Assay (Promega, Madison, WI, US).

To determine the cytotoxicity of MSC-BiTE, the supernatants from MSCs (MSC-BiTE and MSC-EV) were collected. Samples were diluted 5-times with a gradient dilution. The final dilutions of the supernatants were 20 ×, 100 ×, 500 ×. The tumor cells lysed at a dilution of 500 × were observed using an inverted microscope after 20 h' killing. The other experimental details were the same as those described above.

### 2.10 Growth inhibition of HER2-positive tumor xenografts *in vivo*

Female NOD/SCID mice (6–8 weeks old; Charles River, Beijing, China) were randomly divided into two groups (*N* = 8): NCIN87 + PBMC + MSC-BiTE and NCIN87 + PBMC + MSC-EV groups. NCIN87 cells (5 × 10^6^ cells/mouse), PBMCs (1.67 × 10^6^ cells/mouse), and MSCs (1 × 10^6^ cells/mouse) were fixed and subcutaneously implanted into the mice. The tumors and weights of the mice were measured every three days, until the tumor volume reached 1000 mm^3^. The severity of liver damage in mice was determined by elevated levels of serum aspartate aminotransferase (AST) and alanine aminotransferase (ALT; Sangon). Survival curves of different groups were drawn using a tumor volume of 1000 mm^3^ as the criterion.

### 2.11 Measurement of BiTE (HER2/CD3) concentration in mouse plasma

To evaluate the pharmacokinetic (PK) parameters of the BiTE (HER2/CD3) protein, six specific pathogen-free-grade BALB/c mice (20 g, male; Charles River) were divided into two groups (groups A and B; *n* = 3) based on the intensive sampling time. A single dose of 10 μg/mouse of BiTE (HER2/CD3) was administered through the tail vein of the mice. Bleeding from the mice eye socket at appropriate time intervals. For group A, 100 μL blood was collected 0.25, 0.75, 1.25, 3, and 6 h after administration. For group B, 100 μL blood was collected 0.5, 1, 2, 4, and 10 h after administration. Blood was collected in *EP* tubes containing anticoagulants. Plasma was collected *via* centrifugation, and used for the determination of plasma BiTE (HER2/CD3) levels *via* ELISA. A non-compartmental analysis model was employed to determine the pharmacokinetic (PK) parameters with the WinNonlin software.

An *in vivo* experiment was performed to compare the plasma levels of recombinant BiTE (HER2/CD3) antibodies and BiTE (HER2/CD3) secreted by the MSC-BiTE cells. BALB/c mice were divided into two groups (n = 3) and administered with either 2 × 10^6^ cells/mouse MSC-BiTE or 5 μg/mouse of recombinant BiTE (HER2/CD3) antibodies *via* the caudal vein. For MSC-BiTE, 100 μL blood was collected at 4, 24, 72, and 144 h after administration. For BiTE (HER2/CD3) antibodies, 100 μL blood was collected at 4, 6, 10, and 24 h after administration.

### 2.12 Statistical analyses

Data analyses were performed using the GraphPad Prism 8.0.2 software (unpaired *t*-test). Scatter-dot plots depict the means, with error bars representing as mean ± SEM.

## 3 Results

### 3.1 Preparation and purification of BiTE (HER2/CD3) using the 293F expression system

To evaluate the application of BiTE-secreting MSCs in cell-based therapies, we first characterized the cytotoxicity of BiTE. Targeting the HER2 antigen, we constructed a BiTE with the structure HER2VL-HER2VH-CD3VH-CD3VL (Fig. 1A), where VH and VL denote the variable heavy and light chains of anti-HER2 and anti-CD3 antibodies, respectively. A schematic representation of the BiTE structure is shown in Fig. 1C. To determine its efficacy, we expressed BiTE (HER2/CD3) in the mammalian 293F cell line. A 55-KDa protein was expressed and purified using Ni-affinity chromatography (Fig. 1D). Bands in the electrophoretic gel were at the same location under both reducing and non-reducing conditions, indicating that the expressed protein existed as a monomer.

### 3.2 *In vitro* activity of BiTE (HER2/CD3)

We assessed the bioactivity of BiTE (HER2/CD3) in terms of its binding affinity, T-cell recruitment, and cytotoxicity. EC_50_ of BiTE (HER2/CD3) and HER2 antigen protein was 32.51 ng/mL (Fig. 1E), indicating that BiTE (HER2/CD3) had a strong binding affinity to the HER2 antigen. BiTE (HER2/CD3)-mediated T-cell recruitment was further investigated using flow cytometry. The result showed that BiTE (HER2/CD3) group was significantly higher T-cell recruitment (8.5 % CFSE/PKH26 assembly) compared to trastuzumab (1.82 %) or OKT3 (1.27 %) controls (Fig. 1F). Flow cytometry revealed that BiTE (HER2/CD3) specifically redirected T cells to the HER2-positive tumor cells. Next, the cytotoxicity of BiTE (HER2/CD3) was evaluated in tumor cell lines with different HER2 expression levels (Fig. 1G). BiTE (HER2/CD3) exhibited potent cytotoxicity across tumor cell lines with varying HER2 expression levels.

### 3.3 Determination of BiTE (HER2/CD3) expression levels in MSCs

**Fig. 2:**
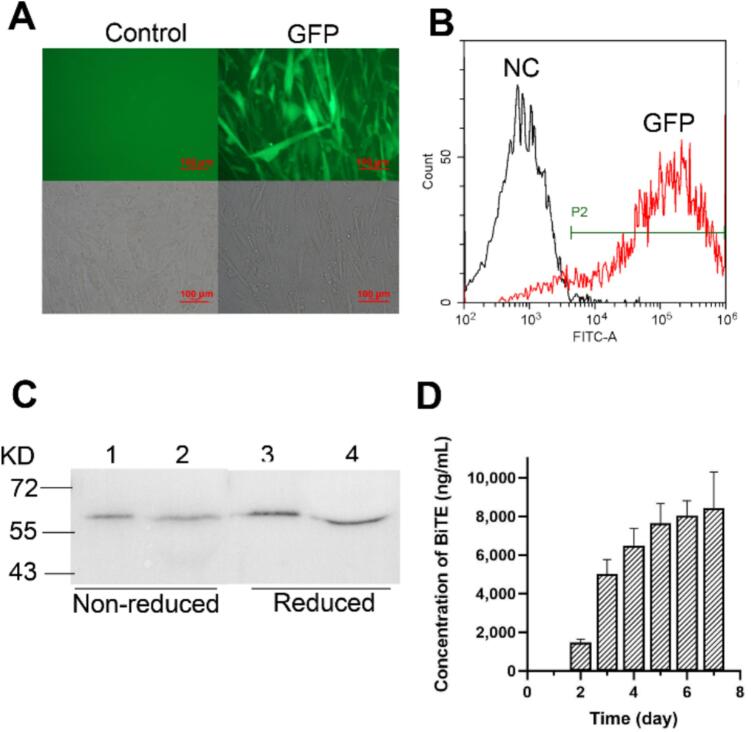
Detection the lentivirus efficiency of infecting MSC and the expression of BiTE (HER2/CD3) in MSC-BiTE. (A) Fluorescence and bright-field images of infected MSC-GFP and control MSC-EV. (B) The transfection efficiency was detected by flow cytometry. (C) The western blot of supernatants of MSC-BiTE. 293F-BiTE, BiTE (HER2/CD3) expressed by 293F cell lines; N, nonreduced; R, reduce. (D) The concentration of BiTE (HER2/CD3) protein in supernatants of MSC-BiTE was detected by ELISA.

We engineered MSC to obtain BiTE-expressing MSCs. Initial attempts using the Neon Transfection System (Invitrogen) yielded low transfection efficiency (<10 %; data not shown). Then we attempted lentivirus transduction of MSCs. The lentivirus, including GFP fluorescent packaging, was processed in HEK293T cells. Green fluorescence was clearly observed in the experimental group, but not in the control group with the empty vector using fluorescence correlation microscopy (Fig. 2A). Flow cytometry analysis demonstrated 85 % transduction efficiency after optimization (Fig. 2B).

Next, the lentivirus containing BiTE (HER2/CD3) was transduced into MSCs. Expression of BiTE (HER2/CD3) in the MSC supernatant was determined by western blotting after 3 days. The positions of the bands were consistent with 293F-expressed BiTE controls (Fig. 2C). To evaluate the yield of BiTE (HER2/CD3) in the infected MSC supernatant, we sampled the cells daily and measured the concentration of the protein using ELISA. The concentration of BiTE (HER2/CD3) peaked at 8000 ng/mL on Day 7 with sustained production through Day 5 (Fig. 2D).

### 3.4 Identification of infected MSCs

Following verification of successful genetic modification of MSCs for BiTE expression, we further evaluated the effect of lentiviral transduction on MSC properties. According to the minimal identification criteria for human MSCs established by the International Society for Cellular Therapy (2006) (Dominici et al., 2006), we verified cell surface markers of native and transduced MSCs (MSC-EV and MSC-BiTE) by flow cytometry. All groups showed positively for CD73, CD90, and CD105, while remaining negative for CD34, CD45, CD11b, CD19, and HLA-DR (Fig. S1). The multipotency of transduced MSCs was confirmed through adipogenic and osteogenic differentiation assays. After adipogenic and osteogenic induction, both MSC-EV and MSC-BiTE groups formed lipid droplets and calcium nodules, respectively (Fig. S2). Cell surface marker detection and adipogenic and osteogenic differentiation experiments illustrated that lentivirus transduction had no effect on MSC phenotypic and functional characteristics.

### 3.5 Inhibitory effects of MSCs on tumor cells

**Fig. 3:**
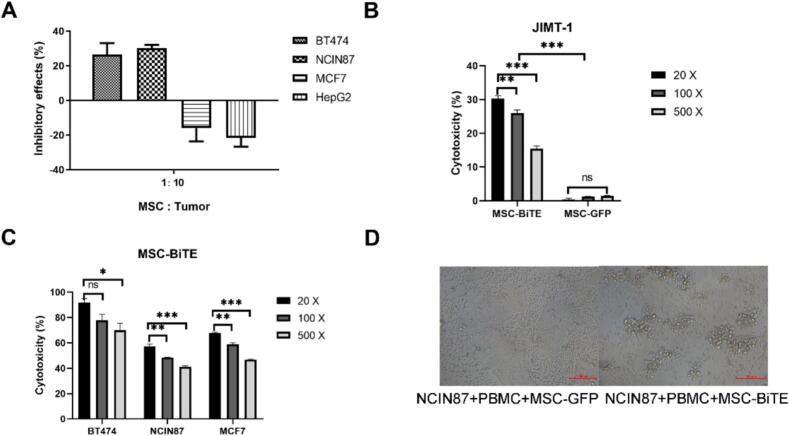
The inhibitory effects and induction death of MSC-BiTE on HER2-positive tumor cells by secreting BiTE (HER2/CD3). (A) The inhibitory effects of MSCs on BT474, NCIN87, MCF7, HepG2 tumor cells. (B) The cytotoxicity of MSC-BiTE on JIMT-1 tumor cells, with the MSC-GFP as the control group. Each experiment was replicated thrice. (C) Dose-dependent killing of MSC-BiTE on BT474, NCIN87 and MCF7 tumor cells. (* *P* < 0.05; ** *P* < 0.01; *** *P* < 0.001. The presented data represent the mean ± SEM from three independent experiments) (D) Tumor cell killing of dilution ratio 500 × about NCIN87 were photographed by an inverted microscope (200 ×). The left is control group MSC-GFP, the right is MSC-BiTE group.

To assess whether MSCs directly influence the growth of tumor cells, we co-cultured MSCs and tumor cells for 72 h and determined their growth-inhibitory effect using the CCK-8 assay. MSCs improved the proliferation of MCF7 and HepG2 cells but inhibited that of BT474 and NCIN87 tumor cells (Fig. 3A).

### 3.6 Cytotoxicity *in vitro*

To examine tumor lysis induced by BiTE (HER2/CD3) in the presence of PBMCs, a cytotoxicity assay was performed. JIMT-1, a HER2-positive cell line, was used as the target at a 10:1 E:T ratio. The supernatants of MSC-BiTE were diluted five times with a gradient dilution, and MSC-GFP treated in the same way served as a negative control. After 20-h incubation, dose-dependent tumor lysis was observed in the MSC-BiTE group (Fig. 3B), whereas MSC-GFP controls showed no cytotoxicity. We also assessed the cytotoxicity of MSC-BiTE against different HER2-expression tumor cells (BT474, NCIN87, MCF7), with all exhibiting concentration-dependent cell death (Fig. 3C). Microscopic analysis revealed that tumor cell lysis with T-effector cell clustering (500× dilution) in MSC-BiTE group. Notably, tumor cells in the control group exhibited intact cell morphology, whereas the T effector cells were scattered (Fig. 3D). These results suggest that the killing and recruiting effects of MSC-BiTE are triggered by its supernatant.

### 3.7 Anti-tumor activity *in vivo*

**Fig. 4:**
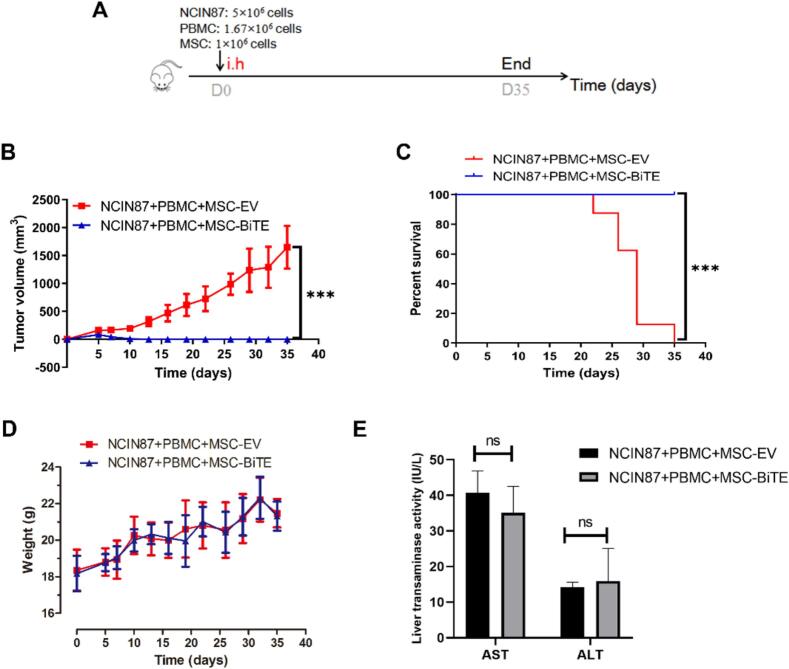
Tumor suppressing effects of MSC-BiTE against NCIN87 tumor in mice. (A) Methodology for the tumor therapy experiment. (B and D) Tumor size and body weights from different groups (*n* = 8) (*** *P* < 0.001). Bar values represent mean ± SD. (C) Changes in the percentages of surviving mice over time (*** P < 0.001). Survival curves were drew using tumor volume of 500 mm^3^ as the criteria. The analysis of progression-free survival time was conducted employing the Kaplan-Meier methodology and Log-rank test. (E) AST and ALT were detected to evaluate mice liver damage. Bar values represent mean ± SEM.

BiTE (HER2/CD3) secreted by modified MSCs has a lethal effect on tumor cells *in vitro*. Next, we tested the efficacy of MSC-BiTE *in vivo*. MSCs, PBMCs, and NCIN87 tumor cells were subcutaneously injected at fixed doses (Fig. 4A). In mice bearing established NCIN87 xenografts, treatment with MSC-BiTE not only controlled tumor growth but also significantly extended the survival compared to that of the MSC-EV group (Fig. 4B and C). MSC-BiTE demonstrated no body weight loss in comparison to the control group (Fig. 4D). To investigate liver damage in mice, AST and ALT levels were measured at the end of the experiment. AST and ALT levels in the MSC-BiTE group were not different from those in the MSC-EV group (Fig. 4E), suggesting that MSC-BiTE has no adverse effects on the liver of mice.

### 3.8 Pharmacokinetic analysis

The BiTE structure enables potent tumor cell killing at low concentrations. However, its half-life is too short for its clinical use. The PK parameters of BiTE (HER2/CD3) were evaluated in mice after a single-dose of 10 μg/mouse *via* tail vein injection (*n* = 3/group). A non-compartment model was applied, and the data showed that BiTE (HER2/CD3) exhibited a biphasic disposition (Fig. S3). The *in vivo* half-life of BiTE (HER2/CD3) protein was 1.47 h in our system, which is consistent with literature values (Ferl et al., 2018; Labrijn et al., 2019).

We compared the *in vivo* plasma levels of BiTE (HER2/CD3) in mice injected with MSC-BiTE cells or BiTE (HER2/CD3) protein. First, we examined the plasma levels of BiTE (HER2/CD3) protein in mice at various time points after tail vein injections of BiTE (HER2/CD3) at a dose of 5 μg/mouse (Table 1). The mice had mean plasma BiTE (HER2/CD3) levels of 445.4 ng/mL after 4 h, 263.4 ng/mL after 6 h, 152.0 ng/mL after 10 h, and 92.5 ng/mL after 24 h. We also determined the plasma levels of BiTE (HER2/CD3) protein in mice after a single intravenous injection of 2 × 10^6^ MSC-BiTE cells. The mice exhibited mean plasma BiTE (HER2/CD3) levels of 181.5 ng/mL after 4 h, 130.9 ng/mL after 24 h, and 93.3 ng/mL after 72 h. Moreover, BiTE was not detected after 144 h. Although modified MSC-BiTE could not achieve plasma levels as high as 5 μg/mouse, it significantly extended the metabolic time of BiTE (HER2/CD3) in mice.

**Table 1:** BiTE (HER2/CD3) concentration in mouse plasma after administration of BiTE (HER2/CD3) or MSC-BiTE cells.

Treatment	Dose	Route	Mean BiTE (HER2/CD3) concentration (ng/mL)			
BiTE (HER2/CD3)	5 μg	iv	*4* *h*	*6* *h*	*10* *h*	*24* *h*
			445.4	263.4	152.0	92.5
MSC-BiTE	2 e^6^ cells	iv	*4* *h*	*24* *h*	*72* *h*	*144* *h*
			181.5	130.9	93.3	0

## 4 Discussion

To our knowledge, this represents the first report utilizing UC-MSCs as delivery vehicles for BiTEs in oncology. Here, infected MSCs secreted BiTE (HER2/CD3) and had an obvious inhibitory effect on tumor growth *in vitro* and *in vivo*. Furthermore, the half-life of BiTE was prolonged in mice.

BiTE is a bispecific antibody with a molecular mass of approximately 55-kDa, which lacks an Fc domain, resulting in rapid clearance (1.25 ± 0.63 h) *in vivo* (Labrijn et al., 2019). While BiTEs show efficacy in hematological malignancies (Frankel and Baeuerle, 2013), their application in solid tumors remains challenging (Lutterbuese et al., 2010). A significant drawback of BiTE antibodies is their short half-life in the plasma. Therefore, prolonging the serum half-life is necessary to maintain for high efficacy of BiTE. Several approaches, such as the addition of HSA or Fc domain (Goldstein et al., 2020; Mandrup et al., 2021; Zuch de Zafra et al., 2019), have been used to solve this problem. Tarlatamab, a DLL3-targeting BiTE incorporating an Fc domain for extended half-life, received FDA accelerated approval in May 2024 for relapsed/refractory small cell lung cancer (SCLC). The clinical evidence supports that half-life extension strategies are critical for achieving therapeutic efficacy of BiTEs in solid tumors. However, a larger molecule would have decreased penetration into the tumor microenvironment (Wang et al., 2019). As delivery systems, MSCs can not only overcome the drawback of a short half-life by continuously secreting BiTEs but also keep the penetration ability of BiTEs.

Furthermore, we demonstrated the feasibility of using MSC as a delivery system. A large number of previous researches have used MSC as delivery systems to tumor microenvironment, and the therapeutic molecule expressed by MSCs included oncolytic viruses, interleukin 12 (IL-12), IFN-β and TRAIL. MSCs can be isolated from bone marrow (BM), adipose tissue, and umbilical cord blood. Although BM was the first source reported to contain MSCs, it may be undesirable for clinical use. The BM was difficult to extract, and differentiation potential of BM-MSC was declined with increasing age (Wang et al., 2016). MSCs isolated from the umbilical cord blood are more acceptable because of their attainability through a less invasive method and their expandability to higher numbers (Kern et al., 2006). Therefore, we used UC-MSCs as a tool for further genetic modification.

Both autologous and allogeneic MSCs present distinct advantages and limitations in clinical applications. Clinical use of MSCs isolated from pathologically affected tissues is contraindicated due to potential functional impairment caused by disease microenvironments (Prakken et al., 2011; Schweizer et al., 2019). For this patient population, allogeneic MSCs presents a superior treatment modality. Compared to autologous MSCs, allogeneic MSCs carry a higher risk of immunogenicity. However, current research indicates that allogeneic bone marrow (BM)-derived and umbilical cord (UC)-derived MSCs may serve as safe and effective therapeutic alternatives to autologous BM-MSCs (Li et al., 2021).

Passage number of MSCs influences the expression of surface receptors during culture, which may lead to cellular senescence and decreased homing capability (Karp and Leng Teo, 2009; Subramanian et al., 2012). Here, we passaged in mesenchymal medium for 30 days, and the cells retained undifferentiated state (data not shown). The cells utilized in this study were obtained within six passages. The cellular expression of surface markers, adipogenesis, and osteogenesis in the cells remained unaltered following infection.

The effects of MSCs on tumor cells remain controversial due to multiple factors, including variations in cell population ratios across distinct animal models, the lesion's location, and administration routes (Klopp et al., 2011). Subramanian et al. reported that BM-MSCs, but not UC-MSCs, differentiate into tumor-associated fibroblasts in the presence of breast and ovarian cancer cells (Subramanian et al., 2012). In our study, we co-cultured tumor cells and MSCs *in vitro* and found that UC-MSCs inhibited the proliferation of NCIN87 and BT474 tumor cells and enhanced the growth of MCF7 and HepG2 cells. This contrasts with reports by Qiao et al., where dermal tissue-derived MSCs inhibited the growth of MCF7 and HepG2 tumor cells *in vivo* and *in vitro* (Qiao et al., 2008a; Qiao et al., 2008b). This discrepancy may be due to the different tissue locations of MSCs. The supernatant of MSC-BiTE also exerted killing effects on NCIN87 and BT474 tumor cells. Therefore, we selected NCIN87 tumor cells for *in vivo* validation. Compared with MSC-EV controls, MSC-BiTE significantly inhibited tumor growth *in vivo* without inducing hepatotoxicity or weight loss in mice. These results highlight the potential clinical applications of engineered MSCs.

## 5 Conclusion

In this study, we developed a novel MSC-based delivery platform utilizing hUC-MSCs engineered for sustained secretion of HER2/CD3 BiTE antibodies. This approach effectively circumvented the pharmacokinetic challenges inherent to conventional BiTE therapies, particularly their rapid plasma clearance. The selection of HER2 as our target antigen was based on its well-characterized amplification and overexpression profiles across multiple solid tumor types. Both MSCs and MSC-BiTE supernatants demonstrated potent cytotoxic activity against HER2+ tumor lines (BT474 and NCIN87). And MSC-BiTE administration resulted in tumor growth inhibition in NCIN87 xenograft model. These results position MSC-BiTE as an efficient therapeutic platform for HER2-positive cancers.

## CRediT authorship contribution statement

**Huifang Zong:** Methodology, Conceptualization, Writing – original draft, Data curation, Visualization. **Xi Li:** Data curation, Methodology, Writing – original draft. **Yunxia Li:** Methodology. **Lei Wang:** Investigation. **Yali Yue:** Methodology. **Jie Chen:** Methodology. **Yong Ke:** Investigation. **Pameila Paerhati:** Investigation. **Lei Han:** Conceptualization. **Yijia Li:** Resources. **Jianwei Zhu:** Writing – review & editing, Funding acquisition. **Baohong Zhang:** Writing – review & editing, Funding acquisition, Conceptualization.

## Ethics approval

This study had approved and adhered to the guidelines of the Institutional Animal Care and Use Committee of the Shanghai Jiao Tong University.

## Funding

This study was partly supported by the 10.13039/501100001809National Natural Science Foundation of China (82073751) and Shanghai Science and Technology Commission Project (20S11904900).

## Declaration of competing interest

The authors declare no competing interests.

## Data Availability

Data will be made available on request.

## Appendix A Supplementary data

Supplementary material Image 1
