# Supplementary material for: Enhance therapeutic efficacy of BiTE (HER2/CD3) for HER2- positive tumors through in vivo expression
Source: Int J Pharm X. 2025 Aug 14;10:100375. doi: 10.1016/j.ijpx.2025.100375 (PMC12410412; doi:10.1016/j.ijpx.2025.100375)
Supplement: Supplementary file 1 — Supplementary material [file mmc1.docx]

**Supplemental Information**

**Enhance therapeutic efficacy of BiTE (HER2/CD3) for HER2- positive tumors through *in vivo* expression**

Huifang Zong^a,b,1^, Xi Li^a,1^, Yunxia Li^a^, Lei Wang^a^, Yali Yue^a^, Jie Chen^b^, Yong Ke^a^, Pameila Paerhati^a^, Lei Han^b^, Yijia Li^c^, Jianwei Zhu^a,b,^*, Baohong Zhang^a,^*

*Correspondence: [bhzhang@sjtu.edu.cn](mailto:bhzhang@sjtu.edu.cn) (B.Z.), [jianweiz@sjtu.edu.cn](mailto:jianweiz@sjtu.edu.cn) (J.Z.)


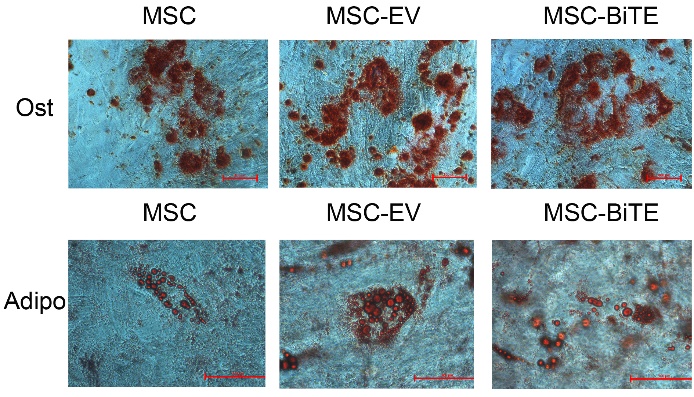


**Fig. S1.** MSCs and modified MSCs (MSC-EV and MSC-BiTE) were able to undergo adipogenesis and osteogenesis under specific differentiating conditions *in vitro*. For osteogenic differentiation, images were captured by inverted microscope using 10X lens, and for the adipogenic differentiation, images were captured by inverted microscope using 20X lens.


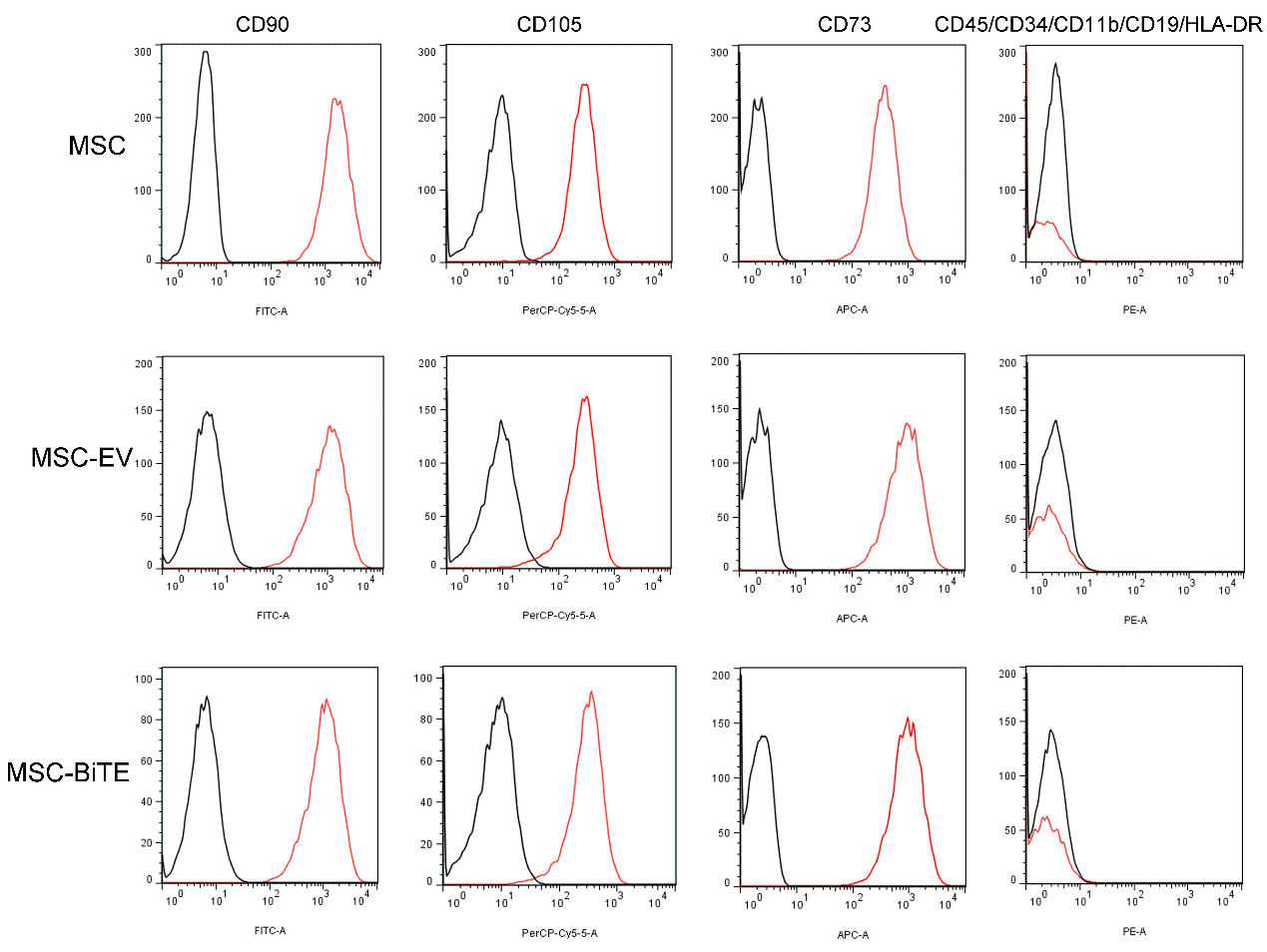


**Fig. S2.** MSCs and modified MSCs (MSC-EV and MSC-BiTE) were positive for the cell surface markers CD73, CD90, and CD105, but negative for CD34, CD45, CD11b, CD19 and HLA-DR as determined by flow cytometry. Black line, negative control; red line, sample cells.





**Fig. S3.** Pharmacokinetic analysis of BiTE (HER2/CD3) antibody. BALB/c mice were injected via the tail vein with 10 μg/mouse BiTE (HER2/CD3). Plasma samples were analyzed by ELISA. The half-life of BiTE (HER2/CD3) antibody in BALB/c mice was found to be 1.47 h. Scatter dot plots depict mean with error bars representing standard error of the mean (SEM) (n =3).
